# The Eye as an Index to Disease

**Published:** 1912-07

**Authors:** Park Howell

**Affiliations:** Atlanta, Ga.


### THE EYE AS AN INDEX TO DISEASE.

By Park Howell, Atlanta, Ga.

The eye offers unusual advantages for the observation of disease symptoms.

The fact that six different cranial nerves lend their supply wholly or in part to this organ makes it a center which is more than apt to be affected whenever there is a lesion of the central nerve system.

The fact that it has a mucous membrane easily accessible for direct inspection, and the existence of transparent media through which the skilled ophthalmologist may see important changes in the eye-ground, the nerve head, and the blood or its vessels, affords an opportunity for observation which is entirely unique.

The eye is such an expressive part of the head that is is our habit when talking with our fellowman to look into his eyes for signs of honest intentions, or symptoms of wavering sincerity.

"An eye like Mars, to threaten and command."

Novelists usually select a hyperope for hero, describing his eyes as bright and flashing.

These rapid and accurate rotations of the ball are symptoms of hyperopia, while in myopia just the opposite occurs.

The Hippocratic faces have the sunken eye which adds no little to this expression of the face when the body is in extremis.

In bodily dissolution, the cornea becomes opaque as one of the signs of death.

A very eminent authority on pellagra has repeatedly called my atention to the appearance of the eye in that affection; it is very expressionless, like the eye of a fish.

In the infectious diseases a *conjunctivitis* in *measles* occurs often earlier than the rash, and in *whooping cough* small subconjunctival hemorrhages are quite obvious. In *cerebro-spinal meningitis* optic neuritis and in *diphtheria* a post-diphtheritic paralysis of cil muscle may confirm an otherwise doubtful diagnosis. A swelling of the lids may mean a kidney involvement or it may be the only sign of an angio-neurotic oedema.

In syphilis an iritis or irido-cyclitis is quite common n the secondary form or a paralysis of one of the extra-ocular muscles (usually the ex-rectus) may be the result of a gumma or a parenchymatous keratitis may indicate the hereditary form, as it does in a large per cent. of cases.

BLOOD VESSELS.

In *mitral disease* an embolism of the central artery may occur with instant loss of sight.

In *aortic regurgitation* the wave or pulsation may often be seen in the retinal arteries.

The changes of *arterio-sclerosis* may be seen in the retinal vessels before the disease is otherwise apparent.

*Leukaemia* may present a characteristic retinal picture, dilated tortuous blood vessels full of pale blood with a pale yellowish fundus.

*Diabetes* may show a retinitis which is sometimes like albuminuric retinitis and sometimes characteristic, but it usually occurs late.

Retinitis of *Brights Disease* is usually well marked, often early and almost pathognominic.

In Freidrich's ataxia and disseminated sclerosis nystagmus is quite common.

In tabes optic atrophy and the Argyle-Robertson pupil. This pupil reaction also in paresis.

In chronic alcohol and tobacco poisoning characteristic changes occur in the color fields before the beginning pallor of the disc.

He would indeed be a bold diagnostician who would say brain tumor unless papillodema or choked disc were present.

In a large percentage of brain lesions the optic tracts and papillary reflex ring carry fibres through such important areas of brain tissue that their localization is facilitated by the changes which occur in the visual fields and the pupillary reflex, e. g., whether the lesion is in front of the chiasm, at the chiasm (as in pituitary tumor) behind the chiasm or above the primary op. centers, may be clearly demonstrated by the combinations in which these symptoms are present or absent, e. g.

In this brief summary I have not attempted to mention all the diseases which may show eye symptoms, nor have I attempted to describe the character of the changes which do occur. But I do hope to have reminded you that there are important eye changes in a great many diseases which the careful diagnostician cannot afford to ignore, and the object of this paper is to plead for more frequent eye examinations.

I do not wish to be understood as advocating an eye examination as a routine procedure, I make no such egregious pretensions. But there are classes of cases which, in my judgment, should *always* have a complete examination and briefly they are about as follows:

1st. Of course, all cases of disease of the eye proper.

2nd. All cases of general disease, exhibiting symptoms referable to the eyes.

3rd. All cases of disease of the central nervous system.

4th. All cases in which the nature of the disease is in doubt.

